# Supplementary material for: A repeated-measures study on emotional responses after a year in the pandemic
Source: Sci Rep. 2021 Nov 30;11:23114. doi: 10.1038/s41598-021-02414-9 (PMC8632939; doi:10.1038/s41598-021-02414-9)
Supplement: Supplementary file 1 — Supplementary Information 1. [file 41598_2021_2414_MOESM1_ESM.docx]

**Appendix to:**

**“A repeated-measures study on emotional responses after a year in the pandemic”**

Table A1. *The 10 most changed LIWC categories in short texts (ranked by BF)*

| **Category** | **BF** | **Cohen’s d (99% CI)** | **Wave 2 vs. wave 1** |
| --- | --- | --- | --- |
| time | 89.90 | 0.34 [0.28 - 0.41]. | + |
| home | 78.14 | 0.32 [0.25 - 0.38]. | - |
| anx | 71.18 | 0.30 [0.24 - 0.37]. | - |
| negemo | 39.41 | 0.23 [0.16 - 0.29]. | - |
| affect | 37.64 | 0.22 [0.16 - 0.29]. | - |
| social | 31.97 | 0.21 [0.14 - 0.27]. | - |
| see | 28.69 | 0.20 [0.13 - 0.26]. | + |
| adverb | 24.71 | 0.18 [0.12 - 0.25]. | + |
| conj | 23.38 | 0.18 [0.12 - 0.24]. | - |
| ipron | 16.39 | 0.15 [0.09 - 0.22]. | - |

Table A2. *Topic terms and differences between phases (short texts)*

| **Topic** | **Cohen’s d (99% CI)** | **Terms** |
| --- | --- | --- |
| Topic 1 | -0.83 [-0.9 - -0.76]. | govern, coronavirus, optimist, lost, know, nhs, made, everi, give, death |
| Topic 6 | 0.75 [0.68 - 0.82]. | get, vaccin, want, just, let, glad, bring, june, sooner, sort |
| Topic 8 | 0.82 [0.74 - 0.89]. | feel, life, anxious, better, day, sad, posit, much, readi, frustrat |
| Topic 13 | 0.78 [0.71 - 0.85]. | thing, time, realli, start, summer, right, make, never, roll, excit |
| Topic 11 | -0.69 [-0.76 - -0.62]. | end, now, year, long, light, way, tunnel, seem, near, last |
| Topic 9 | 0.36 [0.29 - 0.42]. | one, pleas, yet, first, move, dont, respons, case, got, number |
| Topic 3 | 0.36 [0.3 - 0.42]. | peopl, rule, everyon, stay, keep, safe, follow, mask, home, virus |
| Topic 7 | -0.2 [-0.26 - -0.13]. | lockdown, like, come, school, good, month, learn, anoth, thank, littl |
| Topic 14 | 0.29 [0.23 - 0.35]. | see, famili, wait, futur, cant, friend, abl, worri, bore, miss |
| Topic 4 | -0.37 [-0.43 - -0.3]. | will, still, corona, situat, virus, even, ever, whole, chang, open |
| Topic 12 | 0.46 [0.39 - 0.52]. | hope, can, live, soon, restrict, take, freedom, relax, lift, may |
| Topic 10 | 0.24 [0.18 - 0.31]. | normal, back, look, forward, return, now, happi, children, wish, new |
| Topic 5 | -0.2 [-0.27 - -0.14]. | need, pandem, work, mani, health, countri, angri, mental, new, rate |
| Topic 2 | -0.07 [-0.13 - -0.01]. | final, well, enough, done, covid-, vaccin, impact, rollout, effect, fuck |
| Topic 15 | -0.03 [-0.09 - 0.04]. | covid, fed, tire, peopl, world, without, great, complet, put, affect |

Table A3. *The top 20 increased and decreased n-grams for short texts (ranked by the absolute effect size r*)

| **Increased *n*-grams (phase 2 > phase 1)** | |  | **Decreased *n*-grams (phase 2 < phase 1)** | |
| --- | --- | --- | --- | --- |
| *n*-gram | *r* (SE) |  | *n*-*gram* | *r* (SE) |
| vaccin | 0.95 (0.01) |  | stay_home | -0.85 (0.02) |
| covid | 0.83 (0.02) |  | home | -0.81 (0.02) |
| normal | 0.55 (0.02) |  | stay | -0.77 (0.01) |
| back | 0.53 (0.03) |  | worri | -0.69 (0.02) |
| back_normal | 0.51 (0.04) |  | anxious | -0.50 (0.04) |
| end | 0.47 (0.03) |  | pleas | -0.42 (0.04) |
| still | 0.44 (0.03) |  | coronavirus | -0.41 (0.04) |
| look | 0.37 (0.04) |  | safe | -0.40 (0.04) |
| see | 0.35 (0.03) |  | everyon | -0.37 (0.03) |
| lockdown | 0.34 (0.03) |  | virus | -0.33 (0.03) |
| now | 0.30 (0.03) |  | peopl | -0.33 (0.02) |
| want | 0.28 (0.03) |  | situat | -0.32 (0.03) |
| get | 0.26 (0.02) |  | famili | -0.29 (0.03) |
| life | 0.25 (0.03) |  | time | -0.24 (0.03) |
| hope | 0.25 (0.03) |  | live | -0.20 (0.03) |
| thing | 0.21 (0.03) |  | one | -0.16 (0.04) |
| soon | 0.17 (0.04) |  | keep | -0.15 (0.04) |
| like | 0.10 (0.04) |  | need | -0.10 (0.03) |
| rule | 0.10 (0.04) |  | friend | -0.10 (0.03) |
| govern | 0.09 (0.04) |  | go | -0.06 (0.03) |

**A4. Prediction of cluster membership**

The emotion change analysis (Table 3) highlighted two distinct groups of participants (well-coping individuals and individuals who were characterised by resignation). We explore whether the assigned clusters can be predicted from the textual responses provided by the participants. That is, for each participant we train supervised classification models based on textual features that aim to predict the participant’s cluster membership. To achieve this, we consider the long and short texts independently for each phase and train linear classification models using TF-IDF features on a total of four combinations. TF-IDF stands for *term frequency-inverse document frequency* and represents a method to generate numeric features from textual data. For each word and document of consideration, TF-IDF computes the product of the term frequency, i.e., the frequency of a word in a document, and the inverse document frequency, i.e., the inverse of the fraction of documents in which a specific word occurs [1]. Specifically, we featurise the input texts by considering all words occurring in at least 2% of the training set documents and conduct 5-fold-cross validation for each configuration. We examined the performance of Naive Bayes and Support Vector Machine (SVM) classification models. All models were implemented in R and we use the *caret* R package [2] for our implementation. Table 9 shows the performance metrics. We observe that for both short and long texts, and for both waves, the classification performance remains around chance-level (shown in the Base column). This shows that neither of the two models is able to classify the written statements with respect to their cluster membership.

Table A4. *Performance results of Naive Bayes and SVM classification models trained to predict participants’ cluster membership from textual responses.*

| Phase | Long | | | | | | Short | | | | | |
| --- | --- | --- | --- | --- | --- | --- | --- | --- | --- | --- | --- | --- |
|  | Naive Bayes | | | SVM | | | Naive Bayes | | | SVM | | |
|  | AUC | Acc. | Base | AUC | Acc. | Base | AUC | Acc. | Base | AUC | Acc. | Base |
| 1 | 57.23 | 57.36 | 56.42 | 56.50 | 55.77 | 56.42 | 54.76 | 56.54 | 56.42 | 54.24 | 56.13 | 56.42 |
| 2 | 58.87 | 57.95 | 56.42 | 56.68 | 57.71 | 56.42 | 57.88 | 59.25 | 56.42 | 57.32 | 57.83 | 56.42 |

*Note:* Base represents the random guessing baseline for the majority class (averaged over all five folds). AUC and accuracy (Acc.) for each combination are averaged across the five folds.

**References**

1. Jurafsky D, Martin JH. Speech and language processing: an introduction to natural language processing, computational linguistics, and speech recognition. Second Edition, Pearson International Edition. Upper Saddle River, NJ: Prentice Hall, Pearson Education International; 2009.

2. Kuhn M. caret: Classification and Regression Training. 2017. Available: https://CRAN.R-project.org/package=caret
